# Supplementary material for: Single Domain Antibody-Mediated Blockade of Programmed Death-Ligand 1 on Dendritic Cells Enhances CD8 T-cell Activation and Cytokine Production
Source: Vaccines (Basel). 2019 Aug 7;7(3):85. doi: 10.3390/vaccines7030085 (PMC6789804; doi:10.3390/vaccines7030085)
Supplement: Supplementary File 1 [file vaccines-07-00085-s001.pdf]

A

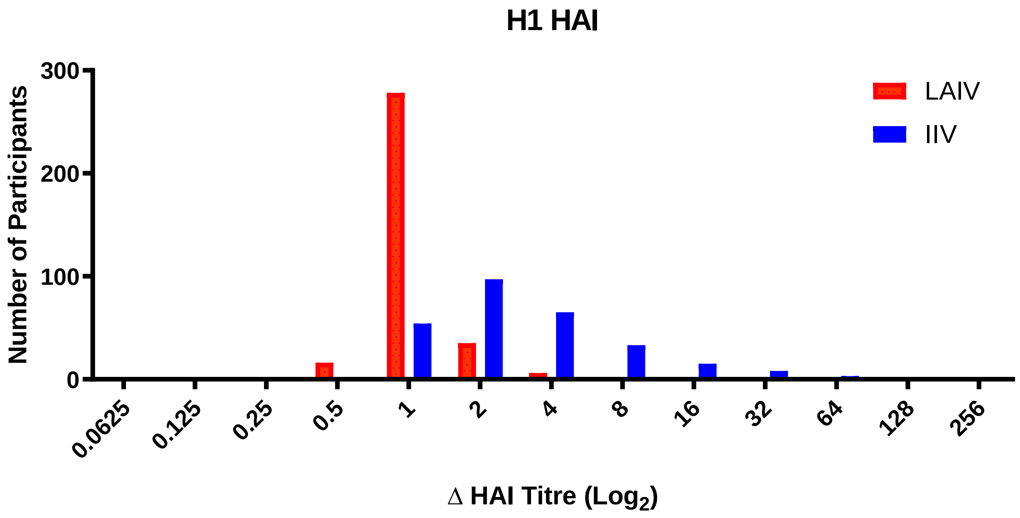

B

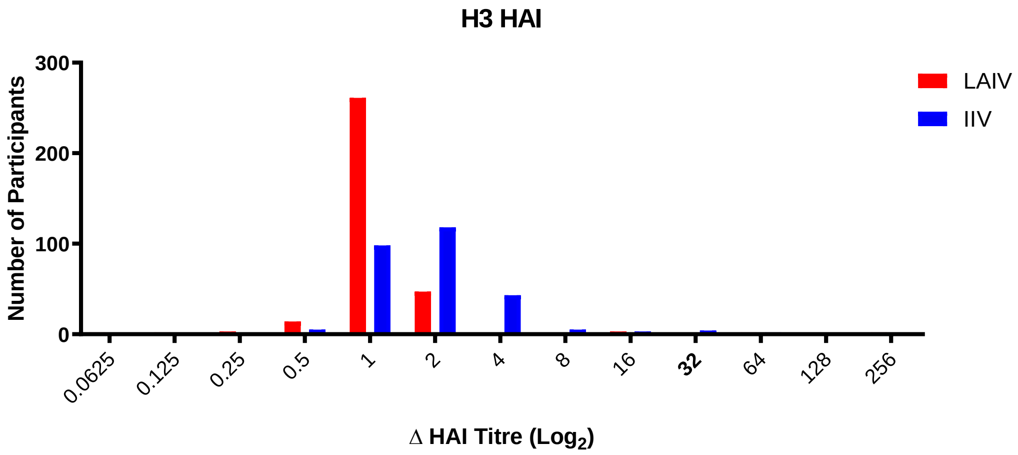

Supplementary Figure 1. H1N1 and H3N2 pre/post-vaccination HAI titer change distribution by vaccine type. HAI assays were performed against (A) Cal/09 H1N1 and (B) Tex/50 H3N2 viruses. Distributions of Log_2 transformed change in HAI titer category from pre-vaccination to post-vaccination were plotted for each subtype. LAIV, n = 340; IIV, n = 278.

A

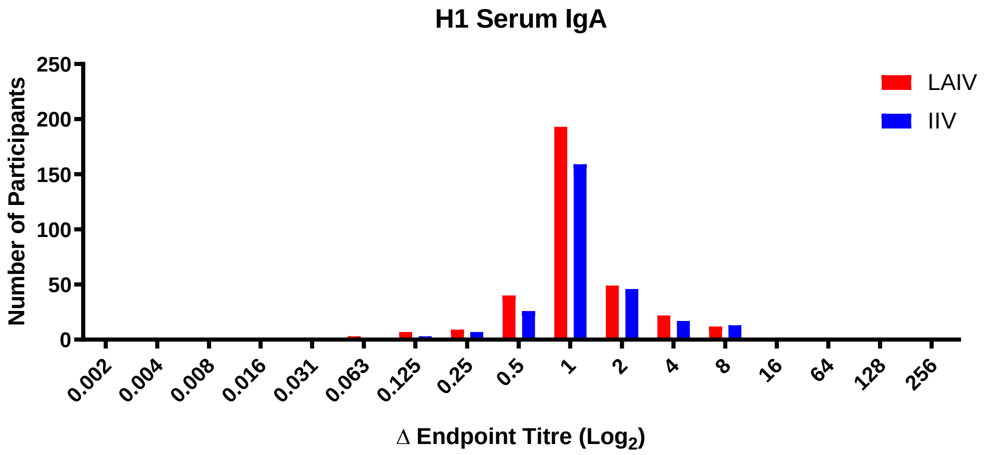

B

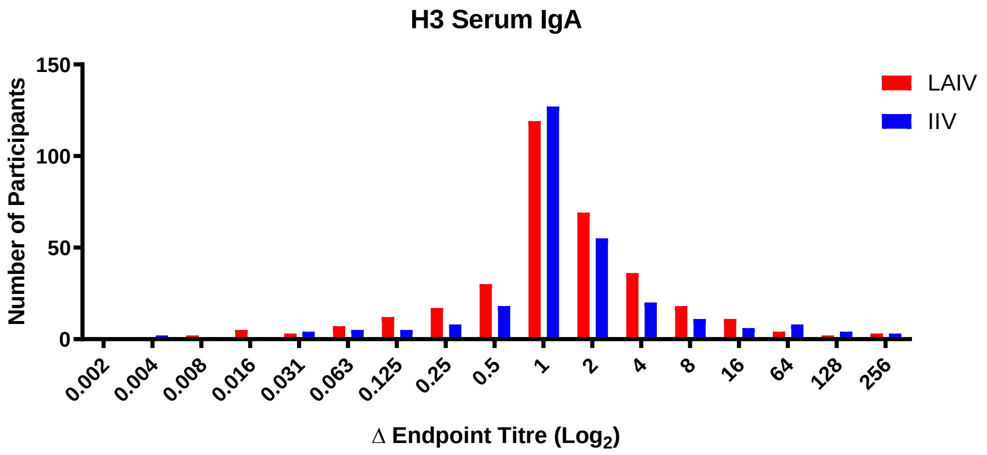

Supplementary Figure 2. H1 and H3 pre/post-vaccination serum IgA endpoint titer change distribution by vaccine type. Endpoint ELISA assays were performed to measure titers of serum-derived IgA using (A) recombinant Cal/09 H1 protein or (B) recombinant Tex/50 H3 protein. Distributions of Log_2 transformed changes in endpoint IgA titer from pre-vaccination to post-vaccination were plotted for each subtype. LAIV, n = 340; IIV, n = 278.

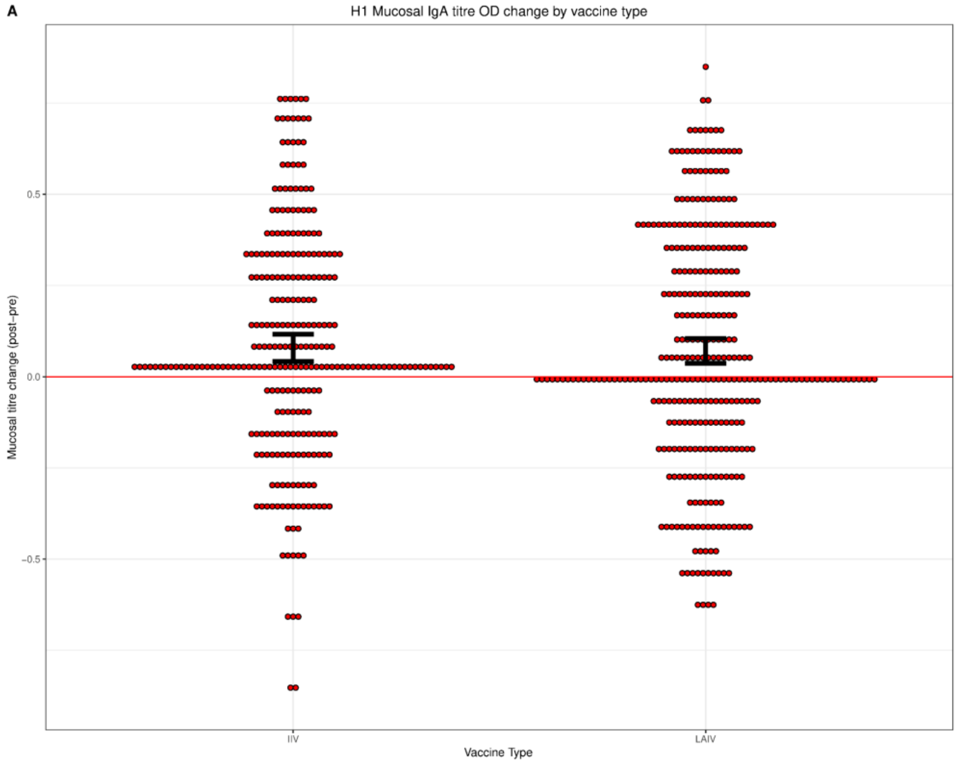

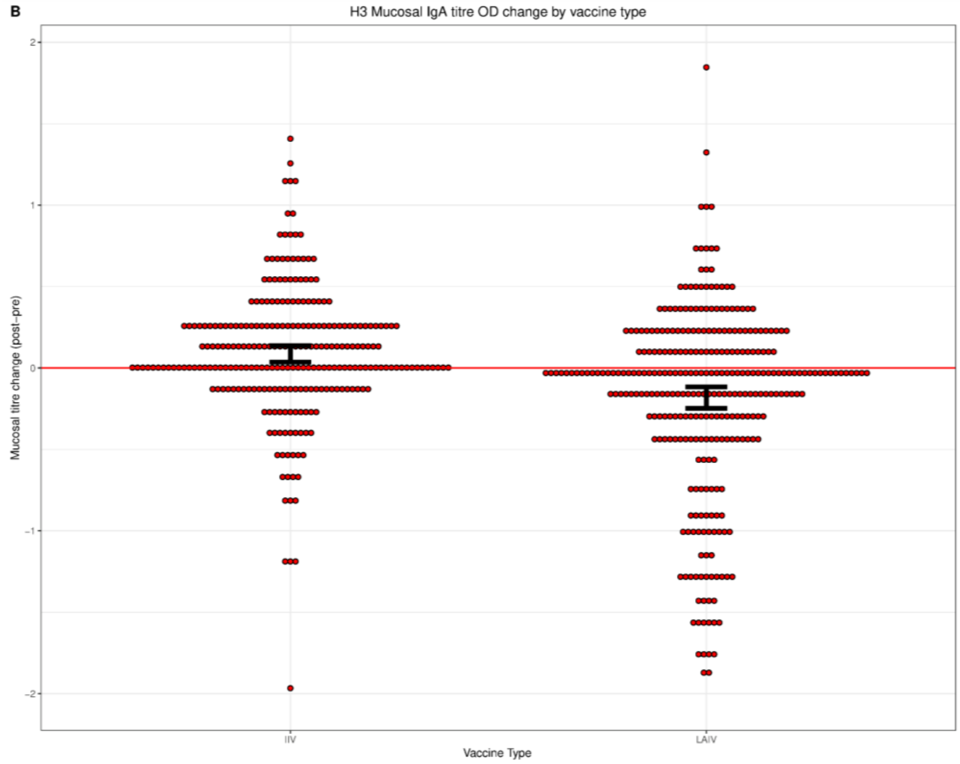

Supplementary Figure 3. H1 and H3 pre/post-vaccination mucosal IgA titer change distribution by vaccine type. ELISA assays were performed to measure titers of mucosal IgA collected by nasal swab using (A) recombinant Cal/09 H1 protein or (B) recombinant Tex/50 H3 protein. Individual data points of normalized optical density from pre-vaccination to post-vaccination were plotted for each vaccine formulation. LAIV, n = 340; IIV, n = 278.

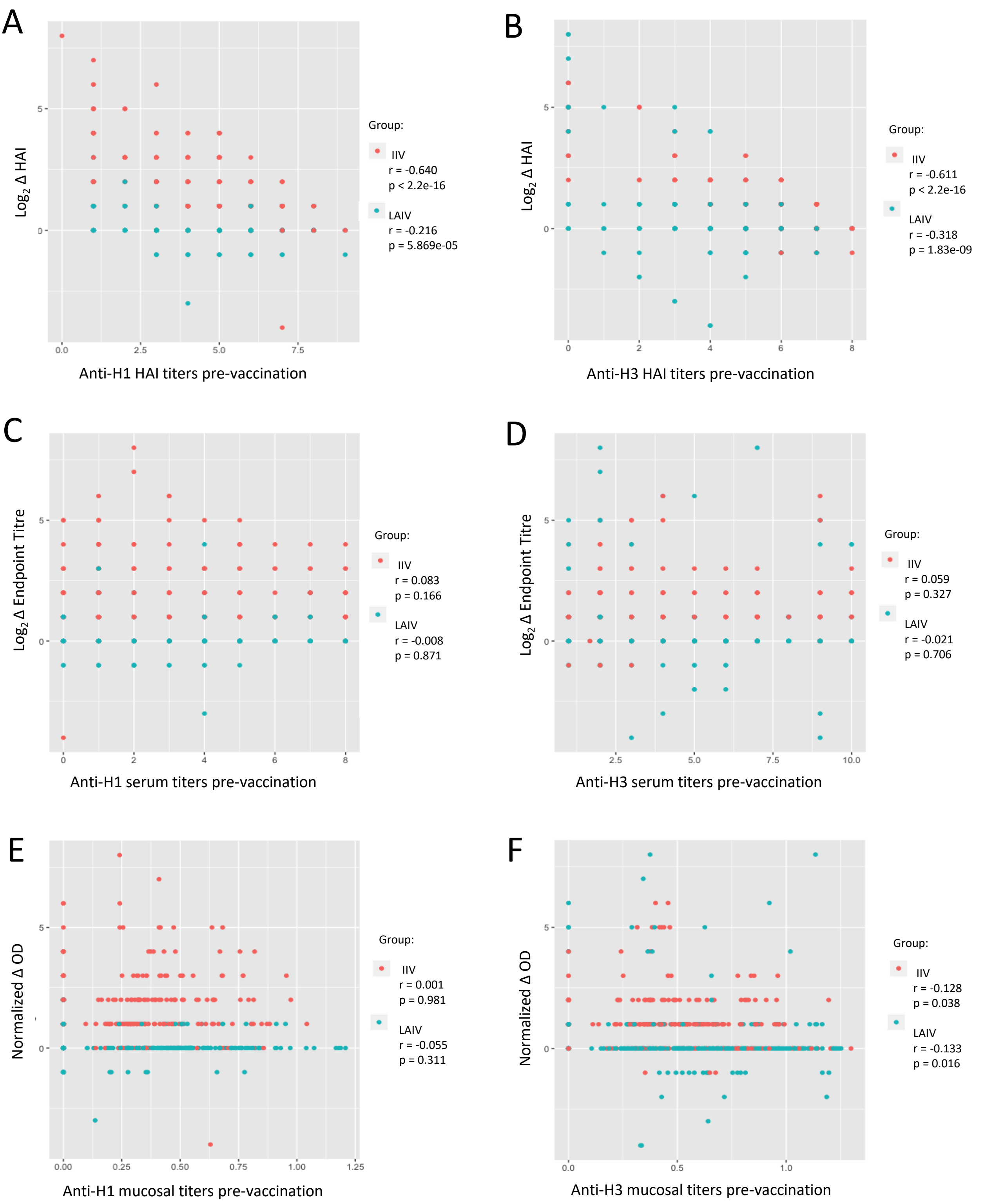

Supplementary Figure 4. Relationship between pre-vaccination antibody titers and post-vaccination response. Pre-vaccination antibody titers against (A, C, E) H1 and H3 (B, D, F) were plotted against change in antibody titers post-vaccination for (A, B) HAI, (C, D) serum IgA and (E, F) mucosal IgA. Statistical analysis was performed using Spearman's rank correlation test.

A

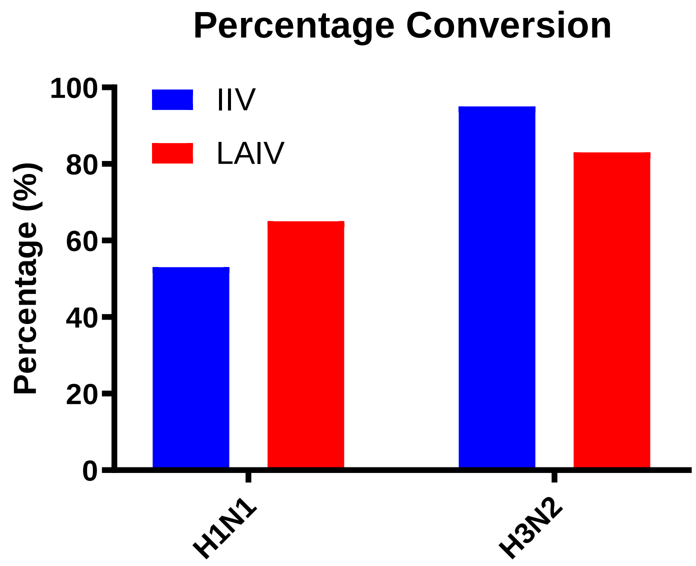

B

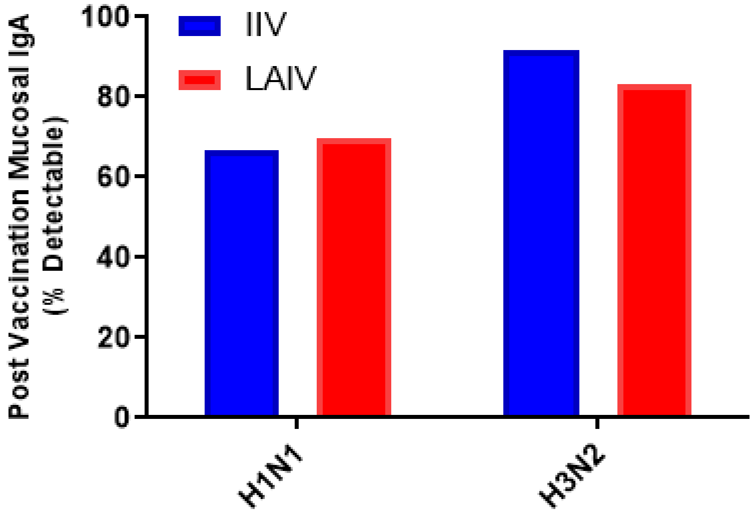

Supplementary Figure 5. Binary quantification of mucosal IgA detection. (A) Percentage of individuals who had no detectable mucosal IgA titers pre-vaccination, whose titers then became detectable post-vaccination (converted). (B) Total percentage of individuals with detectable mucosal IgA post-vaccination.
